# Supplementary material for: Causal Relationship between Aging and Anorexia Nervosa: A White-Matter-Microstructure-Mediated Mendelian Randomization Analysis
Source: Biomedicines. 2024 Aug 16;12(8):1874. doi: 10.3390/biomedicines12081874 (PMC11351342; doi:10.3390/biomedicines12081874)
Supplement: Supplementary file 1 [file biomedicines-12-01874-s001.zip › Supplementary Figure/Figure S8/Leaveoneout plot of MR analysis for TL to ubm-b-1963.pdf]

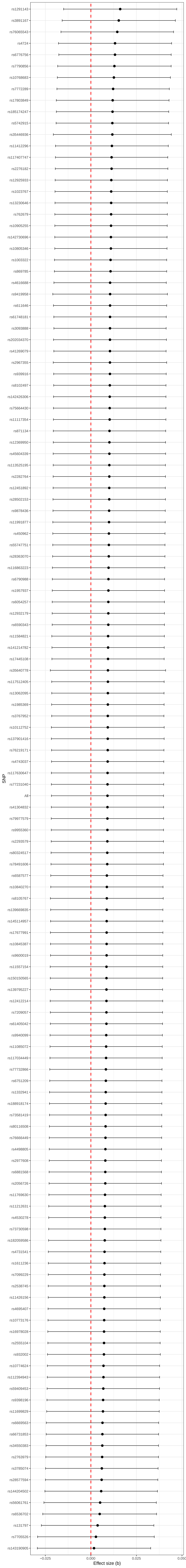
rs1291143
rs3891167
rs76065543
rs4724
rs6776756
rs7790856
rs10768683
rs7772289
rs17803849
rs185174247
rs5742915
rs35446936
rs11412296
rs117407747
rs2276182
rs12925933
rs1023767
rs13230646
rs762679
rs10905255
rs142730696
rs10805346
rs1003322
rs869785
rs4616688
rs9419958
rs611646
rs61748181
rs3093888
rs202034370
rs41269079
rs2967355
rs939916
rs8102497
rs142426306
rs75664430
rs11117354
rs871134
rs12369950
rs45604339
rs113525195
rs2282764
rs12451892
rs28502153
rs9878436
rs11991877
rs450962
rs55747751
rs28363070
rs116863223
rs6790988
rs1957937
rs6054257
rs12932179
rs6590343
rs11584821
rs141214782
rs17445108
rs35640778
rs117512405
rs13062095
rs1985369
rs3767952
rs10112752
rs137901416
rs76219171
rs4743037
rs117630647
rs77231040
All
rs41304832
rs79977579
rs9955360
rs2293579
rs80324517
rs78491606
rs6587577
rs10840270
rs8105767
rs139669835
rs145114957
rs17677991
rs10845387
rs9600019
rs11557154
rs150150565
rs139795227
rs12412214
rs7209057
rs61405042
rs9940099
rs11085072
rs117034449
rs77732866
rs6751209
rs1332941
rs188918174
rs73581419
rs80116508
rs76666449
rs4498805
rs2977608
rs6881568
rs2056726
rs11769630
rs11212631
rs4530278
rs73730598
rs182059586
rs4731541
rs1611236
rs7099229
rs2538745
rs11426156
rs4695407
rs10773176
rs16978028
rs2555104
rs932002
rs10774624
rs112394943
rs59409453
rs9398196
rs11699829
rs6669563
rs66731853
rs34550383
rs2763979
rs3785074
rs28577594
rs144204502
rs56061761
rs6536702
rs131797
rs7705526
rs143190905
SNP
−0.025
0.000
0.025
0.050
Effect size (b)
